# Combinatorial *Atoh1* and *Gfi1* induction enhances hair cell regeneration in the adult cochlea

**DOI:** 10.1038/s41598-020-78167-8

**Published:** 2020-12-08

**Authors:** Sungsu Lee, Jae-Jun Song, Lisa A. Beyer, Donald L. Swiderski, Diane M. Prieskorn, Melih Acar, Hsin-I Jen, Andrew K. Groves, Yehoash Raphael

**Affiliations:** 1 Kresge Hearing Research Institute, Department of Otolaryngology-Head and Neck Surgery, The University of Michigan, Ann Arbor, MI USA; 2 Present Address: Department of Otolaryngology – Head and Neck Surgery, Chonnam National University Hospital, Gwangju, South Korea; 3 Department of Otolaryngology-Head and Neck Surgery, Korea University College of Medicine, Seoul, South Korea; 4 Department of Medical Biology, School of Medicine, Bahcesehir University, Istanbul, Turkey; 5 Department of Neuroscience, Baylor College of Medicine, Houston, USA; 6 Department of Molecular and Human Genetics, Baylor College of Medicine, Houston, USA

**Keywords:** Cell biology, Developmental biology, Neuroscience, Diseases, Medical research, Neurology

## Abstract

Mature mammalian cochlear hair cells (HCs) do not spontaneously regenerate once lost, leading to life-long hearing deficits. Attempts to induce HC regeneration in adult mammals have used over-expression of the HC-specific transcription factor *Atoh1,* but to date this approach has yielded low and variable efficiency of HC production. *Gfi1* is a transcription factor important for HC development and survival. We evaluated the combinatorial effects of *Atoh1* and *Gfi1* over-expression on HC regeneration using gene transfer methods in neonatal cochlear explants, and in vivo in adult mice. Adenoviral over-expression of *Atoh1* and *Gfi1* in cultured neonatal cochlear explants resulted in numerous ectopic HC-like cells (HCLCs), with significantly more cells in *Atoh1* + *Gfi1* cultures than *Atoh1* alone. In vitro, ectopic HCLCs emerged in regions medial to inner HCs as well as in the stria vascularis. In vivo experiments were performed in mature Pou4f3^DTR^ mice in which HCs were completely and specifically ablated by administration of diphtheria toxin. Adenoviral expression of *Atoh1* or *Atoh1* + *Gfi1* in cochlear supporting cells induced appearance of HCLCs, with *Atoh1* + *Gfi1* expression leading to 6.2-fold increase of new HCLCs after 4 weeks compared to *Atoh1* alone. New HCLCs were detected throughout the cochlea, exhibited immature stereocilia and survived for at least 8 weeks. Combinatorial *Atoh1* and *Gfi1* induction is thus a promising strategy to promote HC regeneration in the mature mammalian cochlea.

## Introduction

Hearing loss, one of the most prevalent sensory deficits world-wide, is an incurable disease. In many cases, the cause of hearing loss is the loss of auditory hair cells (HCs) leading to decreased mechano-electric transduction of sound. Loss of HCs can be caused by aging, acoustic overstimulation, ototoxic drugs, genetic factors, or infections^1,2^. HCs are surrounded by supporting cells (SCs), with both cell types originating from a common progenitor cell during development.

In HC epithelia other than the mammalian auditory system, such as fish and birds, lost HCs are replaced by proliferation and/or transdifferentiation of underlying SC. In the zebrafish lateral line, HC damage immediately induces down-regulation of Notch signaling, FGF signaling, and cell cycle exit genes such as *cdkn1b*, which are all important pathways for cell proliferation and differentiation^3,4^. In zebrafish and birds, inhibition of Notch signaling activates *Atoh1*^5,6^, inducing new HC differentiation^7,8^.

In contrast to non-mammalian vertebrates, auditory HCs in the organ of Corti (OC) of mature mammals do not spontaneously regenerate once lost, and therefore hearing loss is permanent. Attempts to induce auditory HC replacement have focused on reprogramming SCs to adopt a HC fate. One route for reprogramming SCs has been via ectopic expression of Atoh1, a basic helix-loop-helix protein that is both necessary and sufficient for HC differentiation, maturation and survival^9–12^. Over-expression of *Atoh1* was shown to produce extra, ectopic and functional HCs in embryonic, neonatal and even adult stages in mouse or rat^10,13–16^. Despite many promising findings, forced expression of *Atoh1* in adult animals showed large variations in regeneration efficiency and functional maturation of HCs^17^ and the outcomes were insufficient for clinical use. The variability and low yield of HC regeneration may be attributed to epigenetic changes in SCs leading to reduced chromatin accessibility^18,19^. The other route for inducing transdifferentiation of SCs is suppression of Notch genes that repress *Atoh1*, such as Notch family molecules. Notch inhibition leads to *Atoh1* activation and HC regeneration in neonatal mice, and to some extent also in the mature ear^20–22^, although once again, the ability of Notch inhibition to promote HC formation declines precipitously with age^23^.

By analogy to attempts at cellular reprogramming in other tissues, *Atoh1* over-expression may need to be augmented by additional transcription factors^24–26^. Such combinatorial reprogramming may be further enhanced by modulating Wnt, Notch or epigenetic modification of hair cell loci^27^. Experiments in transgenic mice demonstrated increased production of new HCs in the adult cochlea by inducing *Atoh1* and either inhibiting *p27kip1* or activating *Pou4f3 or c-Myc*^28–30^, validating the concept of combinatorial gene expression manipulations for enhancing HC regeneration, as recently reviewed^31^.

One candidate for combinatorial manipulation with Atoh1 is Gfi1 (growth factor independence1), a zinc finger transcriptional repressor known to be important for hematopoietic stem cell self-renewal and engraftment^32^. Its *Drosophila* homologue, *senseless,* is expressed with the *Atoh1* homologue *atonal,* and their interaction is required for sensory organ development^33^. In the inner ear, *Gfi1* is expressed in hair cells shortly after *Atoh1* and is required for normal HC development^34^. *Gfi1*-deficient mice initially form cochlear HCs, but they appear disorganized and improperly innervated and eventually all die by P14, revealing that *Gfi1* is important for HC survival. *Gfi1* was shown to be a down-stream target of *Atoh1* and also of *Pou4f3*, another *Atoh1*-regulated gene important for HC survival and maturation^35–37^. More recently, simultaneous over-expression of *Gfi1, Pou4f3* and *Atoh1* in mouse embryonic stem cells markedly induced efficient differentiation of HC-like cells (HCLCs) in vitro^38,39^.

Based on the importance of *Atoh1* and *Gfi1* in HC development and the known interaction between *Atoh1* and *Gfi1* in *Drosophila*^33^, we tested whether *Gfi1* over-expression can enhance cochlear HC regeneration induced by *Atoh1*. We evaluated the combinatorial effect of *Atoh1* and *Gfi1*, both in neonatal cochlear explants and in the adult cochlea of the Pou4f3^DTR^ mouse model, where HCs can be completely ablated by the addition of diphtheria toxin (DT)^40,41^. We show that in both systems, the combination of *Atoh1* and *Gfi1* over-expression produces more HC-like cells compared to *Atoh1* alone.

## Results

### *Gfi1* augments hair cell production by *Atoh1* in neonatal explants

**Figure 1:**
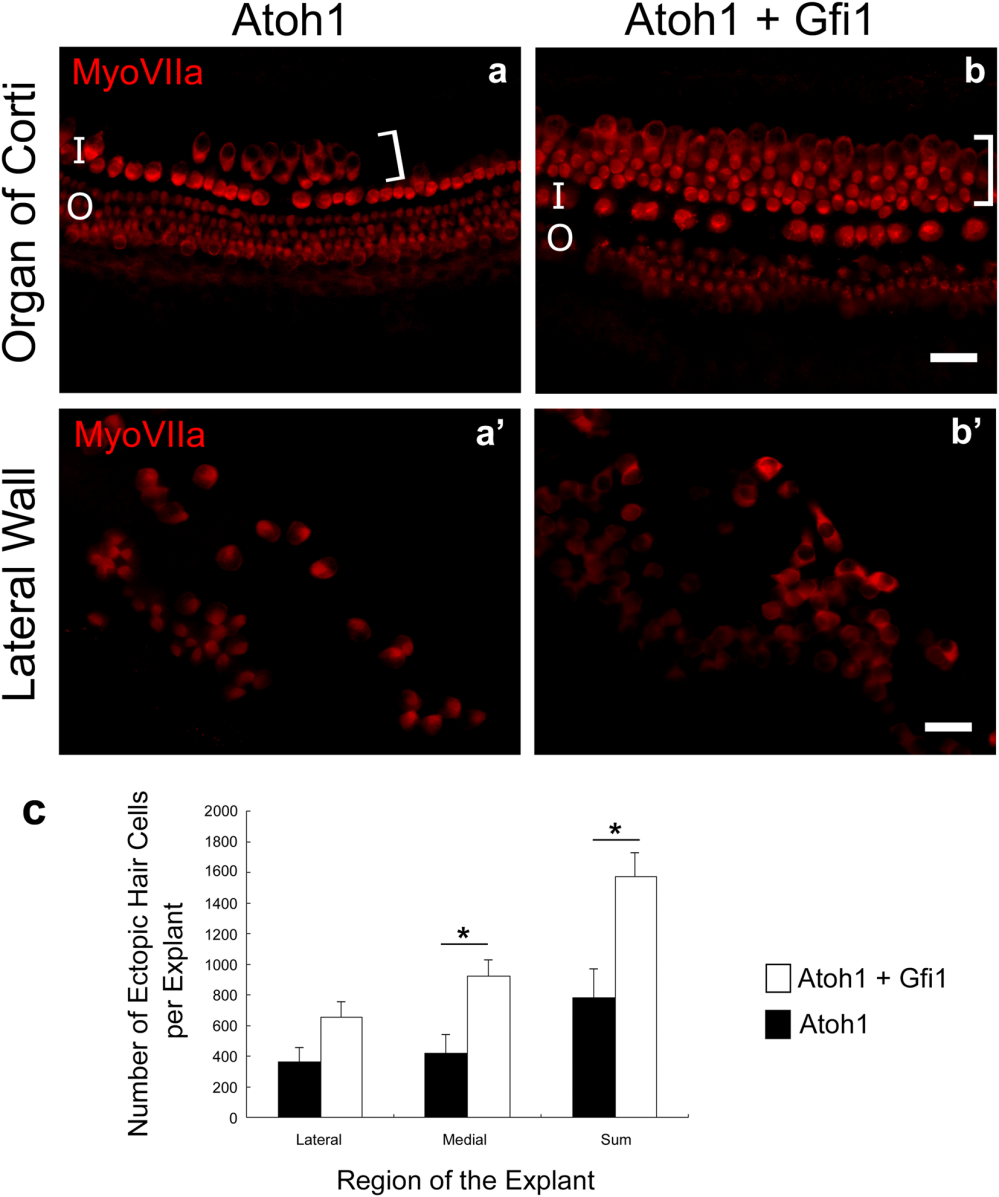
*Gfi1* enhances *Atoh1*-induced ectopic hair cell generation in neonatal cochlear explant cultures. Whole mounts of cultured explants stained for myosin VIIa photographed with epi-fluorescence showing the OC and adjacent areas (**a**,**b**) and lateral wall (**a**′,**b**′). Ectopic HCs (bracket) were induced on the medial side of the inner HC area by *Atoh1* treatment (**a**). Larger numbers of ectopic HCs were observed after *Atoh1* + *Gfi1* treatment (**b**). Ectopic HC induction in the lateral area was evident in *Atoh1* (**a**′) and *Atoh1* + *Gfi1* treatment (**b**′). Statistical analysis showed significantly more ectopic Myosin VIIa positive cells in the region medial to the OC after *Atoh1* + *Gfi1* treatment than after *Atoh1* alone (**c**). *I* inner HC area, *O* outer HC area, *Brackets* ectopic HC. Scale bars represent 30 μm. Error bars are ± SD. Differences between groups were assessed via Student’s *t* test; (*) indicates p < 0.05.

Explanted cochleae obtained from P2–P5 pups and cultured for 6 days without virus showed organized rows of HCs and surrounding non-sensory cells in the area of the OC but did not show ectopic HCLCs adjacent to the OC or in the lateral wall (suppl. Fig. 1a,a′). Similarly, explants treated with *Gfi1* viral vector alone did not display any ectopic HCLCs at the end of the 6-day culture period (suppl. Fig. 1b,b′). This was the case for all explants, suggesting that *Gfi1* by itself is insufficient to induce formation of new ectopic HCLCs in these cultures. In contrast, *Atoh1*-treated explants gave rise to ectopic HCLCs located in the greater epithelial ridge region, medial to the inner HCs (Fig. 1a). Similarly, in cultures treated with both *Atoh1* and *Gfi1* vectors, ectopic HCLCs were found medial to the original row of inner HCs. The number of medially located ectopic HCLCs was 923.3 ± 108.9 in *Atoh1* + *Gfi1* treated explants, which was significantly greater than the 421.8 ± 125.4 in *Atoh1* treated explants (p = 0.007, Fig. 1c) suggesting that co-expression of *Atoh1* and *Gfi1* increased the number of newly generated HCLCs in the medial region of these explants. Ectopic HCLCs were found throughout the explanted cochlear duct, from base to apex.

The lateral wall area in explants treated with *Atoh1* vectors also exhibited Myosin VIIa-positive HCLCs (Fig. 1a′), as did cultures treated with both *Atoh1* and *Gfi1* (Fig. 1b′). Myosin VIIa-positive cells in the lateral wall were located at the apical (luminal) surface of the epithelium, had round nuclei, and a round or oval shape, indirectly suggesting they were originally marginal cells of the stria vascularis that were induced to transdifferentiate to HCLCs. The number of HCLCs observed in the lateral wall area was 364.2 ± 95.6 in *Atoh1* treated explants and 651.8 ± 106.2 in *Atoh1* + *Gfi1* treated explants, which was not significant (p = 0.058, Fig. 1c). Although the difference in number of new HCLCs between treatments was not as large in the lateral wall as in the greater epithelial ridge adjacent to the OC, the total number of new HCLCs in both regions was significantly larger in the *Atoh1* + *Gfi1* treated explants (p = 0.005), suggesting that *Gfi1* efficiently increases the effectiveness of *Atoh1* in inducing differentiation of cells in the cochlea into a HC-like phenotype.

To better characterize the cells generated in the area medial to the inner HCs, we stained cultures with antibodies against prestin, an outer HC-specific marker. Cells in the original outer HC area displayed the typical rings of prestin (suppl. Fig. 2), demonstrating the health of the explants after 6 days in culture, and providing a positive control for the prestin-specific staining. We determined that ectopic HCLCs residing medially to the inner HCs were prestin-negative (suppl. Fig. 2a,b), regardless of their proximity to the inner HC area.

### HC ablation and transgene delivery to supporting cells in mature mice

We next tested whether co-expression of *Atoh1* and *Gfi1* could reprogram non-sensory cells to a HC fate in the mature adult cochlea in vivo. Complete ablation of the original HCs is essential for unequivocal identification of newly-generated cells with HC markers. HC ablation was induced by an intra-muscular injection of DT (20 ng/g) to Pou4f3^DTR^ mice. Ten days after DT, we observed only a few inner HCs remaining in the most apical area. There were no remaining outer HCs in the entire cochlea, showing successful HC ablation by single DT injection (suppl. Fig. 3).

**Figure 2:**
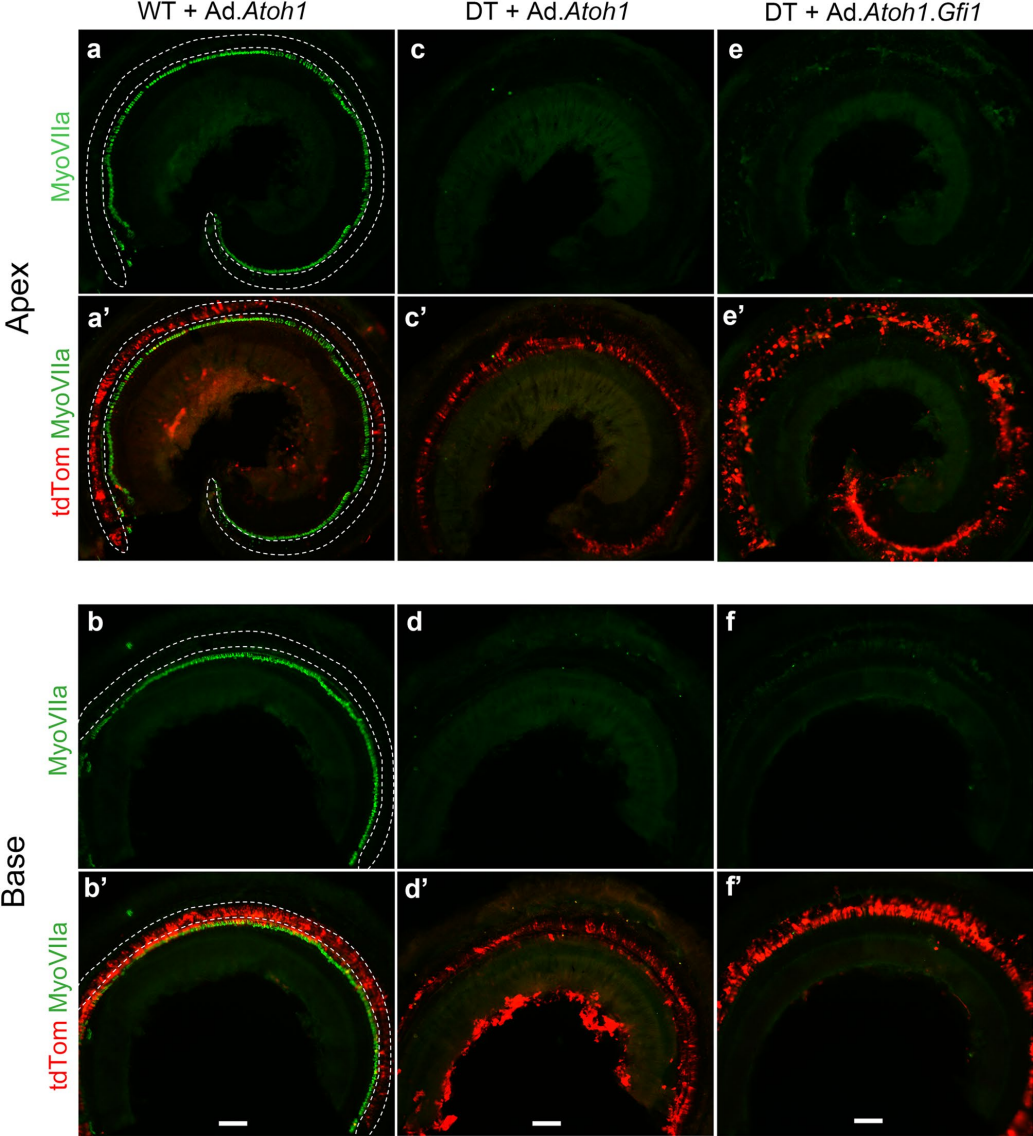
Efficient HC ablation after DT injection and adenovirus scala media inoculation. Images show cochleae of adult mice 10 days after adenoviral inoculation of scala media with or without HC ablation. (**a**,**a**′,**b**,**b**′) Ad.*Atoh1* adenovirus inoculation of wild-type (WT) mouse without DT-induced HC ablation. (**c**,**c**′,**d**,**d**′) Ad.*Atoh1* adenovirus inoculation of *Pou4F3*^*DTR*^ mouse with simultaneous DT injection. (**e**,**e**′,**f**,**f**′) adenovirus Ad.*Gfi1.Atoh1* inoculation of *Pou4F3*^*DTR*^ mouse with simultaneous DT injection. Dotted lines in a, b indicate where OHCs would be found in normal mice. In animals receiving only the injection surgery, most outer HCs are damaged and only a few OHCs remain in the most apical region, but inner HCs are largely intact. In contrast, all HCs both outer HCs and inner HCs, are ablated by simultaneous DT injection in both the Ad.*Atoh1* group (**c**,**d**) and the Ad.*Gfi1.Atoh1* group (**e**,**f**). All groups robustly expressed the tdTomato reporter (**a**′–**f**′). Myosin VIIa, Myosin VIIa. tdTom, tdTomato. Representative figures from 5 biological replicates for each group. Scale bar = 100 μm.

We first investigated the efficiency of viral transduction in the adult cochlea with an adenovirus carrying a tdTomato reporter gene injected into the scala media on the same day as DT injection, as well as into intact ears that did not receive DT. In the latter case (mice that did not receive DT), a severe loss of outer HCs was observed 10 days after the surgery, especially in the base of the cochlea (Fig. 2a,b). This HC loss is due to the mechanical trauma associated with scala media injection, as reported in the past^16,42^. TdTomato was expressed along the OC, especially in the region lateral to inner HCs (Fig. 2a′,b′). Mice that also received DT displayed complete loss of inner HCs and outer HCs (Fig. 2c–f). This result was obtained when adenovirus vectors with *Atoh1-*only or *Atoh1* + *Gfi1* were injected and tissues were harvested 10 days later. In these ears, robust tdTomato expression was seen in OC epithelial cells from the apex to the base in all groups and HCs were absent, confirming that the viruses had no protective effect against DT-induced loss of all HCs (Fig. 2c′–f′). SCs were largely preserved after DT-induced HC ablation, as shown by Sox2 expression (suppl. Fig. 3c,d). SCs were also preserved in groups that had both virus and DT injection (suppl. Fig. 4a–d). When adenovirus was injected into non-HC ablated (DT not injected) cochleae, tdTomato expression co-localized with Sox2-positive SCs (suppl. Fig. 5). The reporter transgene did not co-localize with Myosin VIIa-positive HCs, showing adenoviral scala media injection specifically infects SCs, as previously shown^15,42^. Taken together, we confirmed that DT with or without adenovirus injection can completely ablate HCs. Successful transgene expression was accomplished in SCs in a specific manner by adenovirus injection into the scala media at the same time DT is injected.

### *Gfi1* enhances *Atoh1*-induced cochlear hair cell regeneration

**Figure 3:**
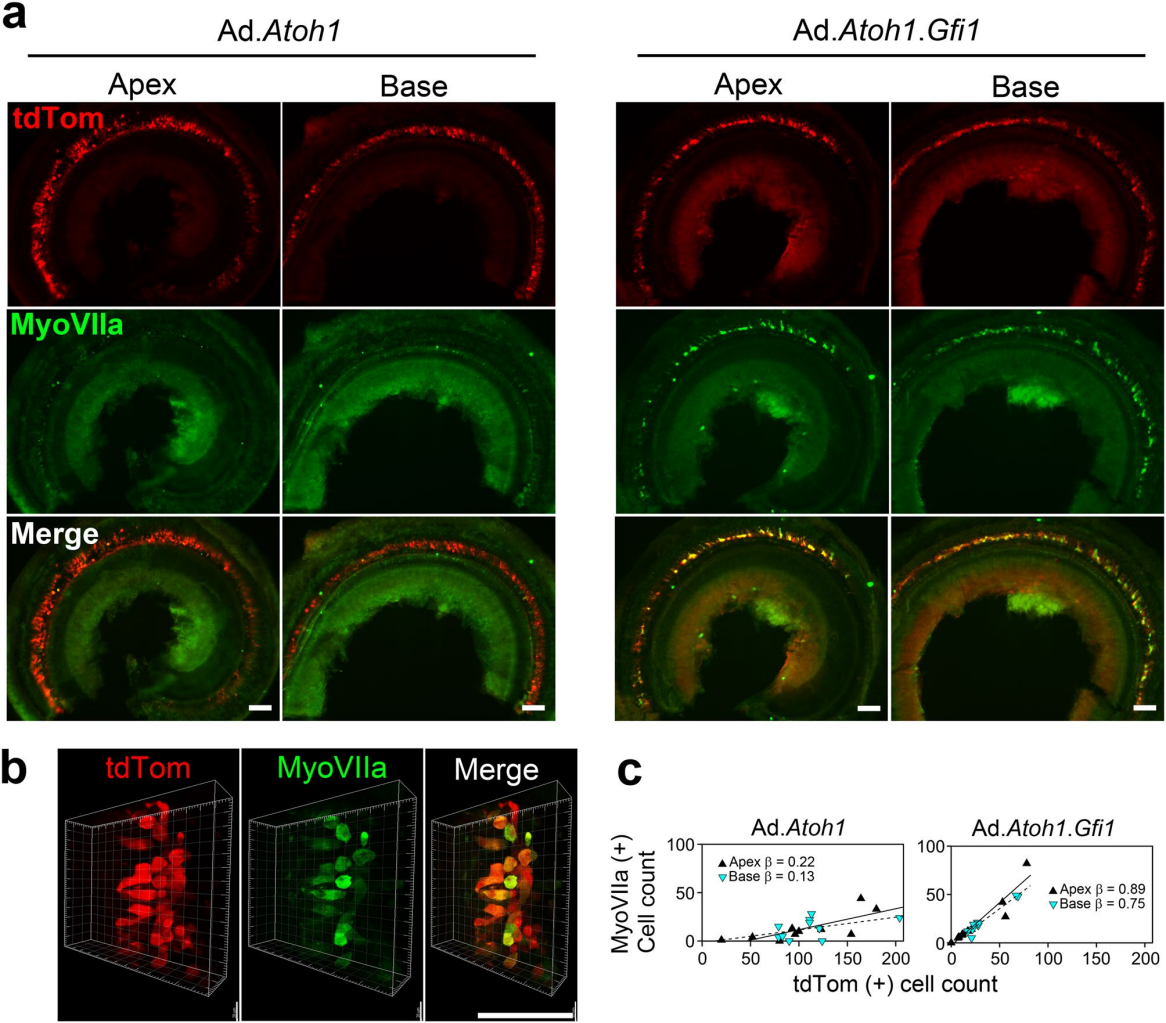
*Gfi1* enhances *Atoh1*-induced hair cell regeneration in the adult cochlea. Images show cochleae of adult mice 4 weeks after DT-induced HC (HC) ablation and adenovirus injection into scala media. The tdTomato (tdTom) reporter is robustly expressed in OC epithelial cells in both cochlear turns (**a**). Mice that received Ad.*Atoh1* had fewer Myosin VIIa positive cells in both apical and basal turn than mice that received Ad.*Gfi1.Atoh1*. Higher magnification images (**b**) show that Myosin VIIa-positive cells are large, variable in shape but usually round, and all co-express tdTom. Linear regression analysis (**c**) shows that the proportion of tdTom-positive cells that were also Myosin VIIa-positive were similar in the apex and base for both treatments and differed between treatments (see text for details). Scale bar = 100 μm.

**Figure 4:**
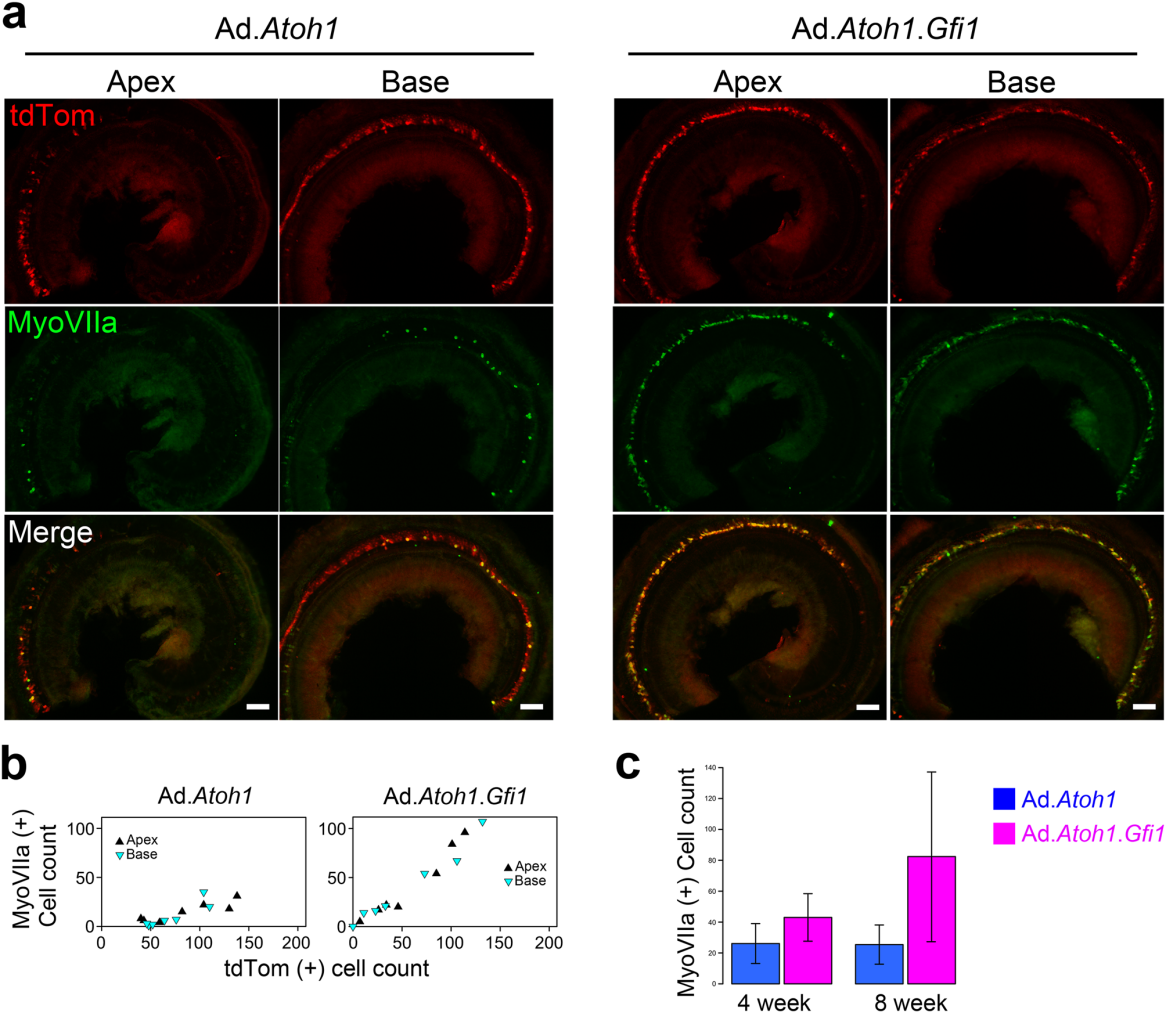
Long term survival of hair cell-like cells. 8 weeks after transgene delivery, there were still numerous Myosin VIIa-positive/tdTomato-positive HCLCs in both treatment groups (**a**). Scatter plots showing counts of Myosin VIIa-positive and tdTomato-positive cells (**b**) demonstrate patterns similar to those seen at 4 weeks; namely, similar proportions tdTomato-positive cells that are Myosin VIIa-positive in the apex and base of each group, and a higher proportion of Myosin VIIa-positive/tdTomato-positive cells in the Ad.*Gfi1.Atoh1* group than in the Ad.*Atoh1* group. The total number of new Myosin VIIa-positive cells was significantly higher in the Ad.*Gfi1.Atoh1* group than in the *Atoh1-*only group (**c**); although difference were not significant due to small sample size at the 8-week time point. Scale bar = 100 μm.

To compare the efficiency of HC generation between adenoviral expression of *Atoh1* alone with *Atoh1* + *Gfi1,* we used mice that were 5–10 weeks old. Immediately following adenovirus inoculation, mice were injected with DT. Ears were collected 4 or 8 weeks later. A comparison was made between the *Atoh1*-only group and *Atoh1 *+ *Gfi1* group, as well as the contralateral ears. At the 4-week time point, new Myosin VIIa-positive cells were found throughout the entire cochlea of mice that received *Atoh1* or *Gfi1.Atoh1* vectors (Fig. 3a). These cells also showed tdTomato expression, confirming that they were derived from SCs that received the adenovirus. In the *Atoh1-*only group, the total number of tdTomato-positive infected cells and Myosin VIIa-positive converted cells were 217.7 ± 78.5 and 26.1 ± 20.4 respectively (Figs. 3c, 4c). In the *Atoh1* + *Gfi1* group, the numbers of tdTomato-positive cells and Myosin VIIa-positive cells were 59.2 ± 32.9 and 43.0 ± 24.3, respectively.

We calculated the percentage of tdTomato-positive cells that were also Myosin VIIa-positive HCLCs, which we termed the conversion ratio. We first analyzed the difference in conversion ratio between apex and base in each group and found that the regression slopes represented by these ratios were not significantly different in either group. In the apex of the *Atoh1-*only group, the ratio was 10.3 ± 7.7%, and in the base, the ratio was 11.4 ± 8.6% (F = 0.064, df = 1 and 17, p = 0.804). In the *Atoh1* + *Gfi1* group, the ratio was 73.8 ± 14.7 the apex of and 66.7 ± 16.1% in the base (F = 1.29, df = 1 and 17, p = 0.272). We then combined the data from the two turns and found that the overall conversion ratio was 11.7 ± 6.7% in the *Atoh1-*only group, and 6.2-fold higher in the *Atoh1* + *Gfi1* group at 72.2 ± 7.6%. The regression slopes represented by these conversion ratios were significantly different (F = 18.5, df = 1 and 17, p = 0.0005). Thus, the apex and base of the mature cochlea responded equally well to reprogramming by both vectors, and co-expression of *Gfi1* with *Atoh1* produced many more Myosin VIIa and tdTomato double-positive HCLCs than *Atoh1* alone*.* The contralateral cochlea which did not receive adenovirus inoculation had no Myosin VIIa-positive cells, nor any tdTomato expression (suppl. Fig. 7). This again demonstrates complete HC ablation by DT injection and that no spontaneous regeneration occurs without the transgene induction. It also demonstrates that the injected virus did not migrate from one ear to the other.

The morphology of new HCLCs varied, some being round, others being more elliptical (Fig. 3b). There were also long, curved cells resembling Deiters cells. The size of the HCLCs was generally larger than that of the original outer HCs. These results suggest that new HCLC are not yet fully mature and that they are derived from SCs.

To determine if the new HCLCs generated by transcription factor reprogramming can survive for extended periods, we examined animals 8 weeks after gene delivery. Similar to the 4-week survival times, there were numerous Myosin VIIa and tdTomato double-positive HCLCs seen in the cochlea after 8 weeks (Fig. 4). The total numbers of tdTomato-positive cells and Myosin VIIa-positive cells were 156.7 ± 52.2 and 25.4 ± 16.8 in the *Atoh1-*only group. The total number of cells for *Atoh1* + *Gfi1* groups were 113.0 ± 89.5 tdTomato-positive cells and 82.4 ± 72.4 Myosin VIIa-positive cells. The *Atoh1*-only conversion ratios were 16.6 ± 5.5% in the apex and 11.8 ± 10.9 in the base, which were not significantly different (F = 0.019, df = 1 and 11 p = 0.893); likewise, the *Atoh1* + *Gfi1* conversion ratios were 68.0 ± 13.8% in apex and 79.8 ± 24.2% in the base and also not significantly different (F = 0.078, df = 1 and 11, p = 0.785). When the data from apex and base at 8 weeks were combined, the conversion ratio was 15.2 ± 8.2% for the *Atoh1-*only group and 69.7 ± 8.5% for *Atoh1* + *Gfi1* group, which was significantly higher than in the *Atoh1-*only group (F = 50.2, df = 1 and 11, p < 0.0001), as it was at 4 weeks. However, the conversion ratio was not significantly different between 4 and 8 weeks for the *Atoh1-*only group (F = 1.958, df = 1 and 14, p = 0.183) or *Atoh1* + *Gfi1* group (F = 0.221, df = 1 and 14, p = 0.646). The change in the total number of Myosin VIIa-positive cells seen at 4 and 8 weeks was not significantly different between the *Atoh1-*only or *Atoh1* + *Gfi1* group (interaction term of two-way ANOVA: F = 2.353, df = 1 and 30, p = 0.136). When the groups were combined, the change in the total number of Myosin VIIa-positive cells seen at 4 and 8 weeks also was not significant (F = 2.198, df = 1 and 30, p = 0.149). These results suggest there was no difference in survival as a result of either transfection or conversion after treatment with either vector (Fig. 4c). Thus, regenerated HCLCs can survive for prolonged periods—at least 2 months—in the adult mouse cochlea.

### Immature hair cell bundles in HCLC

We next tested whether HCLCs (Myosin VIIa-positive/tdTomato-positive cells) induced by Ad.*Gfi1.Atoh1* form stereociliary bundles, using two methods: staining samples with antibodies to TRIOBP, known to be expressed at the rootlet of each stereocilium early in its formation^43^, and scanning electron microscopy (SEM). At 8 weeks after adenovirus inoculation, surviving native HC in non-deafened WT mice displayed a normal arrangement of TRIOBP resembling the arrangement of stereocilia (suppl. Fig. 8a). In contrast, HCLCs induced by the viral vector usually had TRIOBP that was widely distributed in the cytoplasm (suppl. Fig. 8a,c). This pattern was also seen in HCLCs induced in DT-ablated ears (suppl. Fig. 8b). Some unusual HCLC showed streak-like appearance of TRIOBP (suppl. Fig. 8c,c′) revealing an immature state of ciliary development.

**Figure 5:**
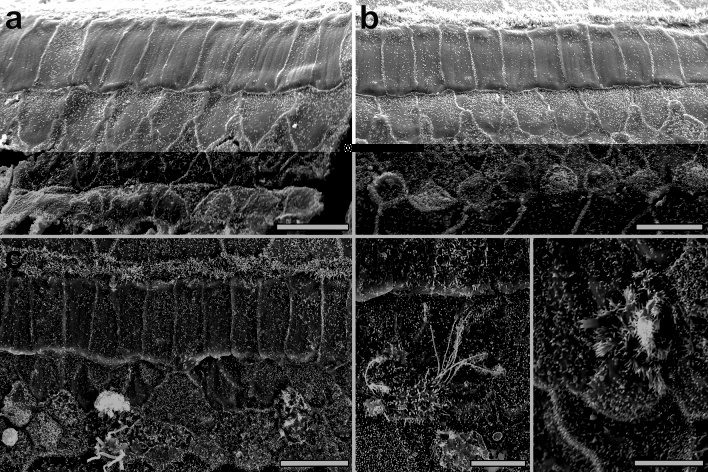
SEM of the organ of Corti in control and Ad.*Gfi1.Atoh1* treated ears. SEM images taken 8 weeks after Ad.*Gfi1.Atoh1* surgery and DT injection in control (contralateral) ear (**a**) or experimental ears (**b**–**e**). Contralateral ear showed no remaining inner and outer hair cell stereocilia. Microvilli on surface delineate rhomboidal shaped cell borders of Deiters cells in the reorganized reticular lamina (**a**). Ad.*Gfi1.Atoh1* injected ear exhibits several round shaped cells bulging from the surface of the reticular lamina and displaying microvilli resembling stereocilia, in a cauliflower appearance (**b**). Some cells are displaying multiple thick kinocilia-like projections arranged in bundles (**c**). Cells with stereocilia throughout the surface with multiple long kinocilia (**d**). Some cells had stereocilia in a clustered form (**e**). Representative images from 3 biological replicates. Scale bar = 10 μm for (**a**–**c**), 5 μm for (**d**,**e**).

SEM analysis performed 8 weeks after injecting Ad.*Gfi1.Atoh1* and DT showed no remaining inner and outer hair cell stereocilia in the contralateral ears (control, right ear, deafened with no further treatment). Instead, we observed typical rhomboidal shaped Deiters cells (Fig. 5a) forming the reticular lamina of deaf ears. In contrast, Ad.*Gfi1.Atoh1* injected ears displayed numerous round cells slightly bulging above the surface of the reticular lamina. These cells displayed short stereocilia in a cauliflower pattern (Fig. 5b). Some parts had cells showing multiple projections appearing like kinocilia (Fig. 5c). Other cells showed stereocilia throughout the apical surface with multiple long kinocilia (Fig. 5d). Some cells had cilia in a clustered form (Fig. 5e). However, none of the new cells displayed normal V-shaped ciliary arrangement.

### Connectivity of HCLCs with neurons

**Figure 6:**
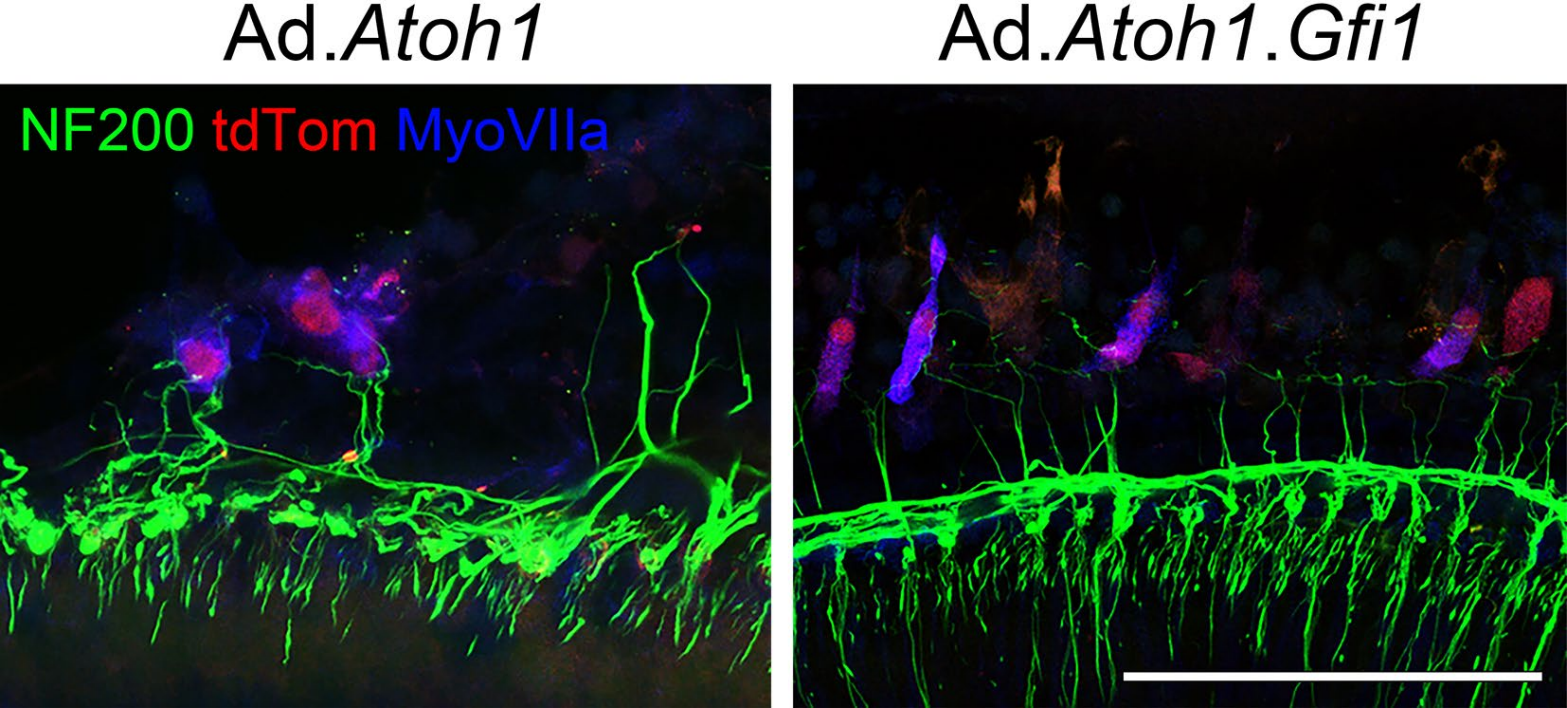
Nerve fibers near regenerated HCLCs. Samples collected at 4 weeks after adenovirus (Ad) and DT-induced HC (HC) ablation. Spiral ganglion neuron (SGN) fibers were visualized by Neurofilament 200 (NF200) staining. Myosin VIIa (MyoVIIa) and tdTomato (tdTom) double-positive cells were associated with nerve fibers along the cell body. Both groups—adenovirus with *Atoh1* (Ad.*Atoh1*) and with *Atoh1* + *Gfi1* (Ad.*Gfi*.*Atoh1*) showed similar association with SGNs. Representative images are shown from 4 biological replicates for each group. Scale bar = 100 μm.

We next investigated the extent of innervation of regenerated HCLCs with spiral ganglion neurons (SGNs). At 4 weeks after adenovirus injection, staining with antibody to NF200 revealed nerve fibers adjacent to many Myosin VIIa-positive/tdTomato-positive cells (Fig. 6). The patterns were similar in *Atoh1-*only and *Atoh1* + *Gfi1* groups. We stained a subset of the ears for the synaptic marker CtBP2 and did not detect any synaptic contacts between the Myosin VIIa-positive/tdTomato-positive cells and the adjacent neurons (data not shown), suggesting that functional hearing should not be expected at this stage. Indeed, no auditory brainstem responses (ABRs) could be elicited in these ears at the peak intensity of the equipment, suggesting HCLCs are not yet contributing to hearing at this stage (data not shown) and further confirming the complete loss of original HCs.

## Discussion

The central importance of the Atoh1 transcription factor in HC differentiation has inspired attempts to use it to drive HC regeneration but results in adult ears have been variable and inefficient. In the present study, we attempted to promote HC regeneration by supplementing Atoh1 treatment with a second HC transcription factor, Gfi1. We demonstrate that combined Atoh1 and Gfi1 over-expression in neonatal cochlear explants induces generation of numerous ectopic HCLCs in the greater epithelial ridge and the stria vascularis, with significantly more cells in Atoh1 + Gfi1 cultures than Atoh1 alone. We further show that viral-mediated over-expression of both transcription factors in SCs of mature deafened *Pou4F3*^*DTR*^ mice induced new HCLCs throughout the cochlea and that these new HCLCs survived for at least 8 weeks.

### Current status of hair cell regeneration in mature adult cochlea

Ectopic expression of *Atoh1* in the embryonic mouse otocyst is able to drive cochlear progenitor cells towards a HC fate, leading to significant numbers of new cells with morphology and electrophysiological properties that resemble *bona fide* HCs (Gubbels et al., 2008). By the time of birth, the cochlea displays a reduced competence to respond to forced expression of *Atoh1*, such that non-sensory cells of the greater epithelial ridge respond readily to *Atoh1* expression, but SCs do not (Zheng and Gao, 2000; Liu et al., 2012; Kelly et al., 2012), despite the fact that they can readily transdifferentiate into HCs after other manipulations such as inhibition of Notch signaling^23,44,45^. The competence of the greater epithelial ridge to respond to *Atoh1* also declines when ears mature, such that no new HCs are formed when *Atoh1* is activated in 3-week old animals (Kelly et al., 2012; Liu et al., 2012).

A more clinically relevant model for HC regeneration involves the use of mature mammals, traumatized ears and a treatment modality that can eventually be used in human subjects. Ectopic HCLCs were seen after Ad.*Atoh1* injection into endolymph of mature guinea pigs (Kawamoto et al., 2003). This experiment paved the way to work on experimentally deafened ears. Viral mediated expression of *Atoh1* in guinea pigs deafened with kanamycin and ethacrynic acid resulted in appearance of numerous new HCs and improvement of thresholds (Izumikawa et al., 2005). We and others attempted to build on these data but discovered a large variability in the deafness model and the experimental outcomes. In contrast, the *Pou4F3*^*DTR*^ mouse model represents a more reliable way to eliminate all HCs. With the *Pou4F3*^*DTR*^ deafness model we can now continue to investigate *Atoh1*-induced regeneration in mature mammals and explore means to enhance it. Approaches for enhancing outcomes of *Atoh1* over-expression in SCs include combinatorial gene expression and/or other means to facilitate transdifferentiation in SCs.

### Approaches for augmenting Atoh1-induced transdifferentiation

The ability of SCs to undergo transdifferentiation to the HC phenotype declines once the cochlea is mature, but the low rate of transdifferentiation seen in several studies that used *Atoh1* gene therapy or Notch inhibition^15,20^, provides an incentive for attempting combinatorial interventions for enhancing regeneration. This approach has been attempted in other organs. For instance, over-expression of *Ascl1* with HDAC inhibitors in retinal Müller glia can give rise to functional neurons after injury in adult mice^46^. In the inner ear HC regeneration field, Wnt activation along with Notch inhibition and epigenetic modulation has been shown to augment transdifferentiation in young mammals^27^. These studies provide the rationale for our attempt to enhance regeneration in adult mouse cochlea with a combinatorial approach. Other combinatorial approaches can be considered, especially those that also involve inducing a proliferative response in SCs, as previously shown by transgenesis^28^ or gene transfer methods^47^.

### How does *Gfi1* enhance SC transdifferentiation?

*Gfi1* is a vertebrate member of the GPS (Gfi1/PAG-3/SENS) family of transcription factors, which are characterized by a zinc finger domain. *Gfi1* has a SNAG transcriptional repressor domain on the N-terminus^48^. *Atoh1* is both necessary and sufficient for neuronal differentiation in the brain and spinal cord^49,50^, and it has recently been suggested that Gfi1 acts to repress neuronal gene networks during HC differentiation^51,52^. Gfi1 function has been best characterized in the hematopoietic system, where it acts chiefly as a transcriptional repressor by recruiting and forming complexes with chromatin modifier proteins such as histone deacetylase (HDAC) 1–3, histone methyltransferase (G9a) and histone demethylase complex^53–55^. Understanding the targets of Gfi1 in hair cells and the specific epigenetic changes promoted by Gfi1 at these loci during HC differentiation may allow their targeting by pharmacological modulators of the relevant chromatin modifying enzymes, leading to a better efficiency of SC reprogramming.

In addition to acting as a transcriptional repressor, it is possible that Gfi1 may also act directly with Atoh1 to positively regulate HC genes. *Senseless,* the *Drosophila* orthologue of Gfi1 is able to interact physically with the Atoh1 orthologue *atonal* and enhance its transcriptional activity, and this physical interaction does not require the *senseless* protein to bind DNA^56^. Recent evidence in mouse embryonic stem cells suggests that Gfi1 protein also may bind to Atoh1 in regions of DNA that have no Gfi1 DNA binding motifs^51^, supporting this ‘Atoh1 co-factor’ function, but confirmation of this role will require better Gfi1 antibodies or mice carrying epitope-tagged Gfi1 protein. Obtaining SCs transfected with *Gfi1* for RNA-seq, ATAC-seq and other sequencing assays may reveal genes with changed level of expression and suggest downstream effectors of this synergistic action of *Atoh1* and *Gfi1*. Recent data suggests that over-expression of *Atoh1* in the mature utricle can render some HC loci more accessible in SCs on the basis of ATAC-seq assays^57^. It is possible that the addition of Gfi1 can further promote this opening of SC chromatin, or that addition of pharmacological modulators of epigenetic writer and eraser enzymes may make SCs more amenable to transcription factor reprogramming.

Because *Gfi1* plays an important role in HC survival and maturation during development^34^, it is possible that it can also enhance survival of new HCs generated by reprogramming of SCs in the mature ear. *Gfi1* was reported as a down-stream target of *Atoh1* and *Pou4f3*^35–37^. However, in addition to the biochemical evidence described above, genetic evidence suggests that Gfi1 plays its role both dependently and independently of *Atoh1* and *Pou4f3*. *Gfi1* null mice show initial HC differentiation from E13.5 to E16, but cochlear HCs then begin to die and are completely absent in the OC by P14, while vestibular HCs survive in *Gfi1* null mice, albeit with abnormal morphology^34^. *Pou4f3* null mice also show initial HC development, but fail to properly mature and elaborate normal stereocilia. Unlike *Gfi1* null mice, they show vestibular HC degeneration as early as E18.5^58^. Expression of *Gfi1* is absent in *Atoh1* null mice, showing that *Atoh1* is upstream of *Gfi1*^34^. Moreover, both *Gif1* and *Pou4f3* have been shown to enhance *Atoh1* HC differentiation in mouse embryonic stem cells^38^. These results suggest that *Gfi1* has unique and additive effects on *Atoh1* in HC differentiation and regeneration.

### Transgene expression in SCs of deaf *Pou4F3*^*DTR*^ cochleae

The *Pou4F3*^*DTR*^ mouse deafness model allows for rapid and complete HC ablation with survival of the SCs^40,41^, but it appears to have a limitation for gene therapy experimentation. We now show that the SCs that survive after HC loss appear refractory to adenovirus transduction. Reporter genes were not expressed following adenoviral injection into scala media performed a week after HC ablation or later, up to at least 4 weeks (data not shown). For this reason, we injected DT and the adenovirus on the same day. It remains to be found why SCs in the *Pou4F3*^*DTR*^ mouse become refractory to adenovirus transduction, and how they differ in this respect from normal SCs (when HCs are present) or SCs in other deafness models which show efficient transfection^15,42,59^. Once we elucidate the changes in SCs in the *Pou4F3*^*DTR*^ mouse, it would likely be possible to design ways for facilitating transgene expression with virus injection given long term after the loss of HCs. A solution to this experimental problem might also be found by designing different viral vectors that overcome this limitation. For now, not being able to transfect SCs in the deafened *Pou4F3*^*DTR*^ mouse after HC ablation limits our ability to test regeneration after long term HC loss, which would be the typical situation in the clinic.

Reporter gene data presented here and in published work show that adenovirus rarely if ever infects HCs^42,59^. This is in contrast to AAV vectors whose main target in the cochlea are HCs, even when injected into perilymph^60^. When using adenovirus, the selective infection of SCs is advantageous not only because it allows testing hypotheses related to transgene expression in SCs, but also, for regeneration studies, because detection of cells with both reporter gene and Myosin VIIa indicates that cells have been transdifferentiated from SCs.

### The distribution of new HCs in explants and in vivo

We show that *Gfi1* can significantly enhance the HC regeneration induced by *Atoh1* in the developing and adult deafened mouse cochlea. We believe this can provide a new strategy to promote HC regeneration. The regenerative capacity we observed was similar throughout the cochlea. The ability to generate new HCs in the basal turn is especially important because in many forms of hearing loss the basal turn is the predominant site of HC loss.

Our results show HC regeneration in neonatal explants and adults in vivo occurred in different regions. Neonatal explant cultures showed ectopic HCs medially to the OC and in the stria vascularis. In the adult cochlea, new HCs appeared in the lateral part of the OC. This difference might be due to the pattern of infection of the virus as well as the chromatin state of SCs, which are immature in the neonate ears. Work with transgenic mice showed that SCs flanking the OC are more likely to undergo transdifferentiation to HCLCs than cells within the OC, Deiters and pillar cells^61,62^. Another study showed that HCLCs can arise in the Deiters cell region^28^. Together, these studies and the current results show that numerous types of epithelial cells in the membranous labyrinth can undergo transdifferentiation into the HCLC phenotype. For transitioning these experimental approaches to clinical applications, it will be necessary to design ways to restrict formation of new HCs to the area where they are needed.

### Transition to clinical work

Showing that *Gfi1* can augment the ability of *Atoh1* to form new HCs in the deaf cochlea is an important step forward, but the limitations and additional obstacles for clinical significance are numerous and substantial. One limitation is the variability in the outcome which is likely a result of differences between surgeries, which are unavoidable especially with the mouse ear being so small and the access to scala media so complex. Interestingly, in the in vitro work which did not involve a surgical procedure the variability was less pronounced. It is possible that the much larger human ear could enable a more uniform surgical approach once a method for injecting into scala media is designed and implemented.

The most important limitation to clinical use is the fact that the numbers of HCLCs was insufficient, and their differentiation and neural connectivity were incomplete by 8 weeks after treatment. The ranges for the numbers of HCLCs were 6–63 cells for *Atoh1*-only and 5–203 for *Atoh1* + *Gfi1*, showing that all ears responded to the treatments but the extent is variable and the numbers are small relative to the number of HCs in a normal mouse. Nevertheless, we have demonstrated that this combinatorial approach is a step toward effective regeneration of HCs.

Future studies will look at longer time points to determine if the new HCs are able to achieve some degree of functional maturation. Studies like combinatorial stimulation of neuronal growth, such as neurotrophin signaling can also be attempted. In parallel, diagnostic tools are needed to verify that HCs are indeed absent in human ears with hearing loss and to determine the state of remaining SCs in these potential candidates for gene therapy.

## Conclusions

Scala media injection of adenovirus can efficiently and specifically deliver transgenes to SCs in *Pou4F3*^*DTR*^ mice when given at the time DT is injected. *Gfi1* significantly enhances *Atoh1*-induced formation of new HCLCs in cultures of developing inner ears in the area of the OC as well as the stria vascularis. In the mature deaf *Pou4F3*^*DTR*^ mouse, regeneration occurs with *Atoh1* alone but the combination of *Atoh1* and *Gfi1* significantly enhances the numbers of new HCLCs. Longer term experiments are needed to examine maturation and innervation of the new HCs and their level of function.

## Materials and methods

### Animals and deafening protocol

For cochlear organ cultures, CD-1 mice (Charles River Laboratories, Wilmington, MA) were bred in-house, and OC and stria vascularis explants were obtained from P2 to P5 pups.

For in vivo experiments, we used *Pou4F3*^*DTR*^ mice (P*ou4f3*^*tm1.1*(*HBEGF*)*Jsto*^/RubelJ; JAX Stock number #028673) of either gender^63^. The characterization and genotyping of this strain have been described previously^40,63,64^. The colony was maintained by mating transgenic males with CBA/J wild-type females. Mice were 5–10 weeks old at the onset of experiments.

Diptheria toxin (DT; List Biologicals Lab. Inc. cat. #150) was dissolved in saline, aliquoted and stored at − 20 °C until use. Repeat freeze–thaw cycles were avoided. A single dose of 20 ng/g DT was administered intra-muscularly to adult mice. Mice receiving adenovirus (Ad) inoculation surgery, were given DT immediately after the surgery. High-calorie diet gel (DietGel 76A, ClearH_2_0) and an extra bottle of water were provided for the 2 following weeks. Body weight and general health metrics were monitored every day for a week after DT administration.

### In vitro cochlear explant experiments

Freshly harvested cochleae were cultured (37 °C, 5% CO_2_) on collagen gels in 1 ml of DMEM (Invitrogen Corporation, Carlsbad, CA) with N1 supplement (Sigma, St. Louis, MO) and 10 U/ml penicillin G (Sigma). After 3 h, an additional 2 ml of culture medium was added to each dish to submerge the explants and adenovirus vector was added to the culture medium. After 24 h, culture media was replaced to remove viral particles remaining in the medium, and cultures were maintained for 6 days in vitro (DIV). Medium was changed every other day. Controls were incubated with no addition of any viral vector.

Experimental groups received viral vectors encoding *Atoh1*, *Gfi1*, or both. The *Atoh1* vector was Ad.*Math1*.11D (GenVec, Inc., Gaithersburg, MD, USA), used at a final concentration of 4 × 10^9^ PU/ml. The *Gfi1* vector was Ad.*Gfi1*.dlE3#1 (a gift from Dr. Hugo Bellen, Baylor College of Medicine, Houston, TX) used at a final concentration of 4 × 10^10^ PU/ml. When used in combination, the concentration of each viral vector was designed to be identical to that used singly. The *Atoh1* group consisted of 9 animals, *Gfi1* treatment group had 8 animals, and the *Atoh1* + *Gfi1* group had 13 animals.

### Viral vector preparation and scala media injection surgery for in vivo study

Production of the adenovirus vectors used in the in vivo work was initiated based on the results of the culture work and took several years to complete. The vectors we used for the current report were produced by the Baylor College of Medicine Gene Vector Core. One vector carried a tdTomato reporter, the mouse *Atoh1* gene and mouse *Gfi1* gene (Ad.*Gfi1*-*Atoh1-*tdTomato), the other did not include *Gfi1* (Ad.*Atoh1-*tdTomato). Adenovirus serotype 5 (Ad5) was produced from an AdenoX vector that was modified to drive gene expression under the control of the Ef1a promoter. Briefly, an expression cassette containing the two or three genes was put into the pL-ICPI-Ef1 (Addgene: 73355) vector using the TaKaRa In-Fusion cloning system. To achieve co-expression of multiple genes, the coding regions were separated by a picornavirus T2A sequence. The expression construct was sub-cloned into the pAdenoX vector using the restriction enzyme site PI-SceI and I-CeuI. The modified pAdenoX vectors were then used to transfect 293T cells. Validation of successful expression of each gene of interest in the 293T cells was performed using Western blotting and immunofluorescence imaging of infected cells as previously reported (Jen et al., 2019). The viral titers were between 5 × 10^11^ and 5 × 10^12^ VP/ml. In vivo expression of tdTomato and Atoh1 protein was confirmed by immunostaining (suppl. Fig. 6). Although Gfi1 expression was confirmed in 293T cells by Western blot, available Gfi1 antibodies do not work well on adult ear tissue, and so expression could not be verified in vivo. Nevertheless, since Atoh1 and tdTomato are both translated downstream of Gfi1 in our vector, we assume Gfi1 protein is also correctly expressed in vivo, as it is in vitro.

Before the surgery, virus was mixed with Fast Green dye (working concentration 0.025%, Sigma F7252). Viral solution was aspirated into a glass pipette (20 µm diameter), using a Nanojector III (Drummond, 3-000-207) held by a micromanipulator (MM-33, Sutter instrument).

Adenovirus was inoculated into the scala media (endolymph) via a cochleostomy. Mice were anesthetized with Ketamine (120 mg/kg, West-Ward Pharm. Corp., NJ, USA) and Xylazine (7 mg/kg, X-JECT E, Henry Schein Inc., NY, USA) by intra-peritoneal injection. Ketoprofen (5 mg/kg) was given by subcutaneous injection. Mice were kept on a heating pad (K-MOD 100, Baxter) to maintain body temperature throughout the surgery. After shaving and disinfecting with povidone iodine swabs, each mouse was placed in the left lateral decubitus position with a custom-made head holder. An infra-auricular skin incision was made, neck muscles were dissected, and the greater auricular nerve was sacrificed to improve visualization. The tympanic bulla was exposed with a surgical drill (20,000 rpm, Z500, NSK) with 0.5 mm burr tip. After the cochlea was exposed, the mucosa on the middle turn was peeled off with a fine pick. The dark pigment of the stria vascularis was used as an indicator to target the scala media (endolymphatic) compartment. Using a 0.25 mm burr tip, a small hole was made on the lateral bony wall of the middle turn. A glass pipette containing the viral solution was placed in front of the membranous labyrinth. The pipette was advanced 150 µm by a micromanipulator, puncturing the membranous labyrinth and placing the pipette tip inside the scala media. A Nanojector III using a pre-programmed cycle (50 nl/cycle, 5 nl/s speed, 50 s delay, total 300 nl) was used to inject the viral solution, and diffusion into the scala media endolymph was visualized by the Fast Green dye. Once the injection cycle was completed, the glass tip was removed and the injection site sealed with tissue adhesive (Vetbond, 3M). The skin was sutured with 6-0 nylon. Immediately after this step, animals in the deafened group received DT. After recovery, mice were moved to the animal facility. Post-operative Ketoprofen was given for 2 days and the suture site, as well as general health metrics were monitored for 7 days. Sutures were removed after 7–10 days.

### Cochlear whole mount and immunostaining

For in vitro explant cultures, samples were harvested at 6 DIV, fixed in 4% paraformaldehyde for 2 h followed by rinsing in PBS.

For the in vivo experiments, mice were sacrificed, the temporal bones removed, and cochleae were collected. A local perfusion was performed with 4% paraformaldehyde through the round window and a hole made at the helicotrema. Temporal bones were immersed in 4% paraformaldehyde for 2 h for fixation, then rinsed with PBS. Cochleae were dissected into 2 pieces, apex and base. The lateral wall was separated from the OC. Samples were permeabilized with 0.3% Triton X-100 in PBS for 10 min. Non-specific binding of secondary antibodies was blocked with 5% normal donkey serum in PBS for 30 min.

Tissues were reacted with primary antibody, rinsed, and incubated with the secondary antibody. Primary antibodies used in the study were rabbit polyclonal anti-Myosin VIIa (1:50, Proteus Biosciences Inc., #25-6790), mouse mAb anti-Myosin VIIa (1:200, Santa Cruz, sc-74516), rabbit polyclonal anti-RFP (1:200, Rockland, unconjugated 600-401-379S; biotin conjugated 600-406-379), goat polyclonal anti-Prestin (1:200, Santa Cruz, sc-22692), goat polyclonal anti-Sox2 (1:200, Santa Cruz, sc-17319), mouse mAb anti-neurofilament 200 (1:200, Sigma-Aldrich, N5289), Rabbit polyclonal anti-TRIOBP (1:200, Sigma-Aldrich, HPA019769) and mouse mAb anti-CtBP2 (1:300, BD Bioscience, 612044). Rabbit polyclonal anti-Atoh1 antibody (1:100) was a kind gift from Jane Johnson in UT Southwestern (Q863). Secondary antibodies were Alexa Fluor 647 conjugated donkey anti-goat IgG (1:500, Invitrogen), Alexa Fluor 647 conjugated donkey anti-mouse IgG (1:500, Invitrogen) and Alexa Fluor 594 or 488 conjugated donkey anti-rabbit IgG (1:500, Invitrogen). Streptavidin conjugated with Alexa Fluor 594 (1:500, Invitrogen, S11227) was used to detect biotin-RFP primary antibody. Samples were mounted with Prolong Gold (Invitrogen) and kept in a dark box until they were viewed with fluorescence microscopy.

### Epifluorescence and confocal microscopy

Samples were first examined using a Leica DMRB epifluorescence microscope (Leica, Eaton, PA, USA) and recorded with a CCD Cooled SPOT-RT digital camera (Diagnostic Instruments, Sterling Heights, MI, USA). Digital images were obtained in monochrome mode then colorized with Adobe Photoshop version 20.0.0. Samples were then analyzed on a confocal microscope (Leica SP8, Leica, Eaton, PA, USA) and LAS X imaging software was used to obtain Z-stack images.

### Scanning electron microscopy (SEM)

Mice were anesthetized and perfused systemically with 2% glutaraldehyde (Electron Microscopy Sciences, Hatfield, PA, USA) in 0.15 M cacodylate buffer. Temporal bones and bullae were removed, and cochleae collected. Cochleae were further incubated in the same fixative for 2 h. After micro-dissection to expose the OC, the tissues were processed with OTOTO method for SEM. Tissues were dehydrated in graded ethanol solutions, critical point dried and then mounted on a stub using silver paste. Images were taken with a TESCAN Rise scanning electron microscope (https://www.tescan.com).

### Auditory brainstem response

Auditory brain stem responses (ABRs) were assessed 8 weeks after the experimental procedures. Mice were anesthetized with Ketamine (120 mg/kg) and Xylazine (7 mg/kg) by intra-peritoneal injection and kept on a water circulating heating pad (K-MOD 100, Baxter). ABRs were recorded in a sound-shielded chamber (CA Tegner AB, Bromma, Sweden). Tucker Davis Technologies system III hardware and SigGen/BioSig software (TDT, Alachua, FL, USA) were used. Sterile needle electrodes were inserted in both the infra-auricular region and the vertex of the skull. Tone burst stimuli (15 ms duration, 1 ms rise/fall times, 10 per second) were given by closed field. Up to 1024 responses were collected and averaged for each stimulus level at each of the frequencies: 4, 12, 24 and 48 kHz. The stimulus level, starting from 90 dB, was either elevated or decreased by 10 dB steps. The hearing threshold was defined as the lowest stimulus where a response wave was observed.

### Statistical analysis

In the in vitro experiments, the number of ectopic cells positive for Myosin VIIa was acquired using the image analysis software tpsDig2 (version 2.12, State Univ. of NY). Ectopic HCs were grouped according to their location: medial to the inner HCs or lateral to the outer HCs. The total number of ectopic HCs in an explant was acquired and analyzed.

To quantify outcomes of Ad.*Atoh1* and Ad.*Gfi1.Atoh1* vector administration in deaf ears in vivo, we first identified SCs that had received the virus by tdTomato expression. We then counted tdTomato-positive SCs that had been converted to HCLCs based on Myosin VIIa expression. Cell counting was done by a researcher that was blind to each sample’s identity. Quantitative comparisons of counts are reported as mean ± the standard error of the mean. Conversion rate was defined by Myosin VIIa-positive per tdTomato-positive cells. We compared the conversion rate between Ad.*Atoh1* and Ad.*Gfi1.Atoh1* as well as apical versus basal turn in each group. Comparisons were also made between the 4- and 8-week survival groups. For the 4-week time point there were 10 mice in each group. For the 8-week time point, there were 7 mice in each group*.* Ratios like conversion rate are not normally distributed and cannot be compared by conventional statistical methods; however, they are equivalent to the slopes of regressions and those can be analyzed by conventional statistics, namely by ANCOVA. Because treatment effects might differ between turns due to vector diffusion from base to apex and damage due to surgery in the basal turn, we tested for differences between apex and base before pooling data from the two turns to test for differences between treatments. Similarly, when testing whether conversion affected survival, by comparing the proportion of converted cells surviving for 8 weeks to the proportion that was seen at 4 weeks, we first tested for a difference between apex and base at each time point. To determine whether the number of converted cells present at 4 and 8 weeks differed between vectors, we used a two-way ANOVA. These statistical analyses were performed in R version 3.6.2, using the aov function. The p value of the F-ratio was used to judge statistical significance.

### Study approval declaration

Animal studies described in the paper were reviewed and approved by the University of Michigan Institutional Animal Care and Use Committee (IACUC). All methods were performed in accordance with the relevant guidelines and regulations, as follows. The University of Michigan is fully accredited by the American Association for Accreditation of Laboratory Animal Care (AAALAC), and the animal care and use program conforms to the standards set in The Guide for the Care and Use of Laboratory Animals, which includes regular periodic surveillance of animal facilities, review of all funded projects for humane use of animals, and the appropriate use of surgical anesthesia, analgesics and tranquilizers. All animal care facilities are under the supervision of AAALAC-accredited veterinarians.

## Supplementary information

Supplementary Figures.

## Abbreviations

ABR: Auditory brainstem response
DT: Diphtheria toxin
DTR: Diphtheria toxin receptor
Gfi1: Growth factor independence 1
HC: Hair cell
HCLC: Hair cell like cell
OC: Organ of Corti
SC: Supporting cell
SEM: Scanning electron microscopy
SGN: Spiral ganglion neuron
WT: Wild type
