# Supplementary material for: Combinatorial Atoh1 and Gfi1 induction enhances hair cell regeneration in the adult cochlea
Source: Sci Rep. 2020 Dec 8;10:21397. doi: 10.1038/s41598-020-78167-8 (PMC7722738; doi:10.1038/s41598-020-78167-8)
Supplement: Supplementary file 1 — Supplementary Figures. [file 41598_2020_78167_MOESM1_ESM.pdf]

Combinatorial *Atoh1* and *Gfi1* induction enhances hair cell regeneration in the adult cochlea

Sungsu Lee, Jae-Jun Song, Lisa A. Beyer, Donald L. Swiderski, Diane M. Prieskorn, Melih Acar, Hsin-I Jen, Andrew K. Groves, and *Yehoash Raphael

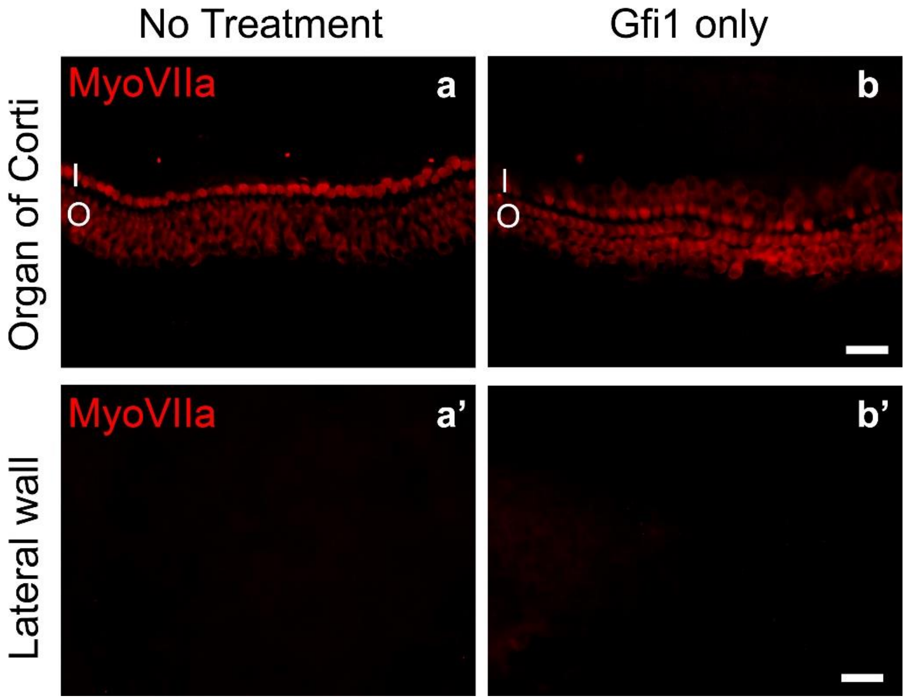

Supplementary Fig. 1. *Gfi1* alone does not generate ectopic hair cells in mouse neonatal cochlear cultures. No ectopic HCs were seen next to the OC in the samples receiving no viral vector (a) or *Gfi1* alone (b), nor were ectopic HCs evident in the lateral wall after these treatments (a’, b’). MyoVIIa, Myosin VIIa. Scale bars = 30 μm.

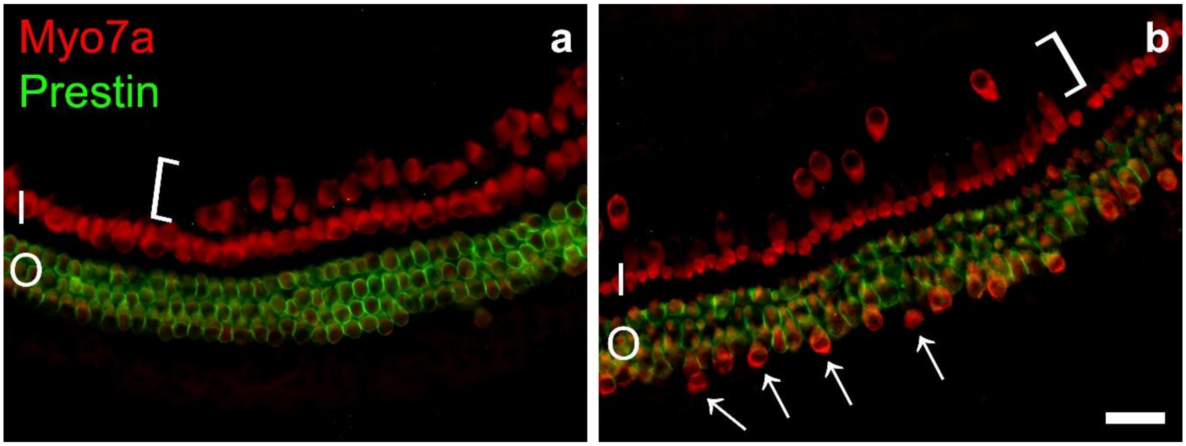

Supplementary Fig. 2. Prestin expression of ectopic hair cells in neonatal cochlear explant cultures. Naïve outer HCs showed strong prestin expression. Inner HCs are devoid of prestin expression. The ectopic HCs observed in medial area after *Atoh1* treatment were negative for prestin immunostaining (a, b; Brackets). Ectopic HCs emerging lateral to 3rd row of outer HCs also showed negative prestin expression (b; arrows). I; inner HC area, O; outer HC area, Scale bars = 30 μm.

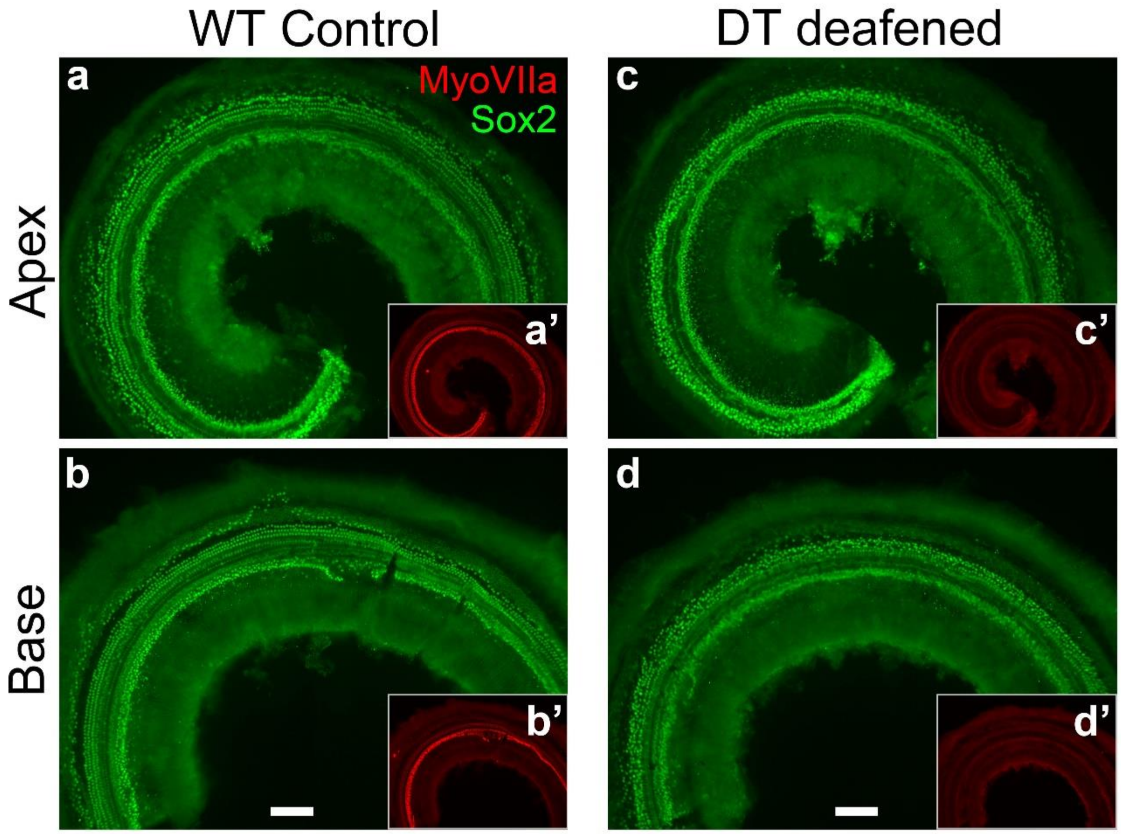

Supplementary Fig. 3. Preservation of supporting cells after DT-induced hair cell ablation

Samples stained for Sox2 and Myosin VIIa obtained 2 weeks after DT injection from wild-type (normal) ears (a, a’, b, b’) and DTR mice (c, c’, d, d’) show that SCs were not eliminated after DT injection (right panels), compared to non-deafened cochlea (left panels). Representative images of 5 biological replicates from each group are shown. MyoVIIA, Myosin VIIa. Scale bar = 100 μm.

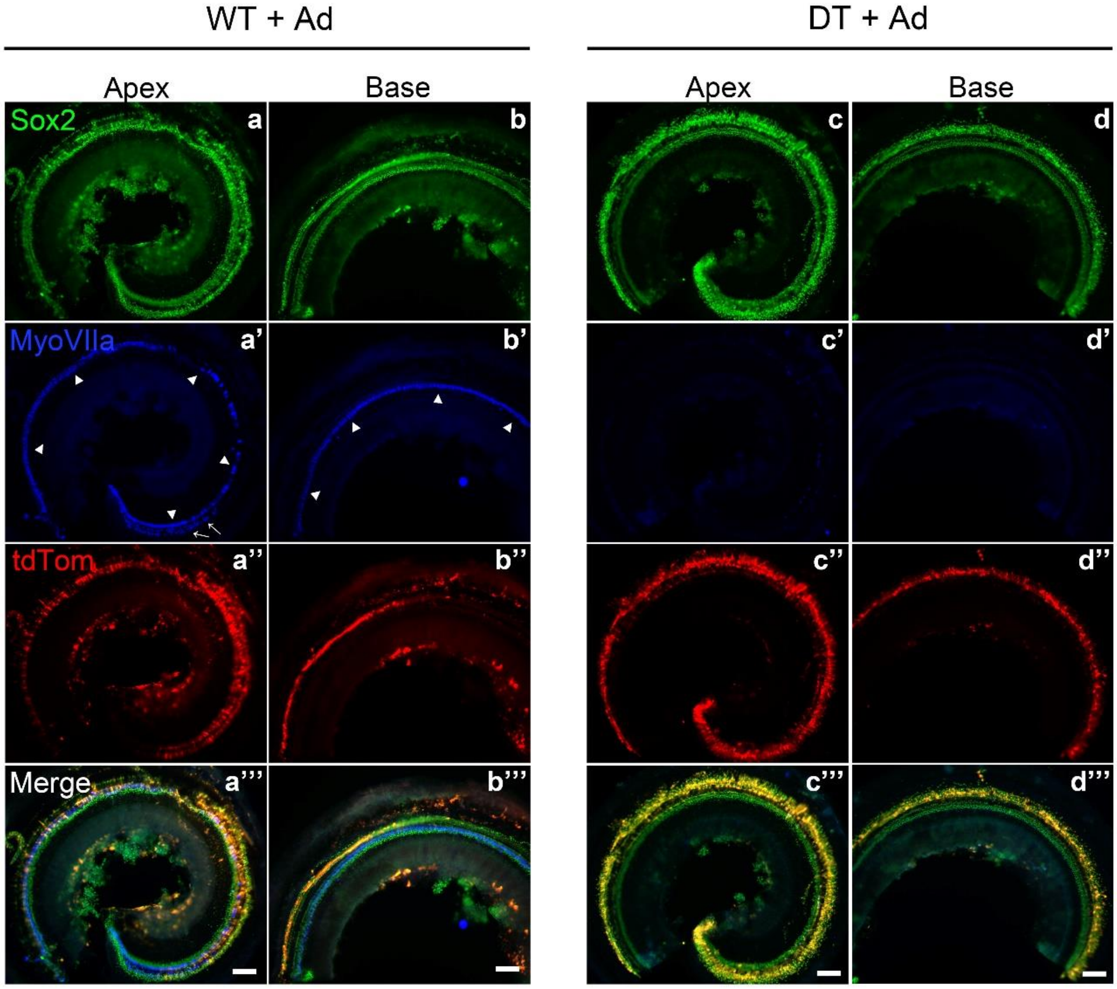

Supplementary Fig. 4. Preservation of supporting cells after adenoviral injection with DT-induced hair cell-ablation. Non-ablated (WT) ears (column a-a’’’ and b-b’’’) exhibit tdTomato (red) in SCs (green for Sox2) while outer HCs (MyoVIIa in blue) are mostly missing especially in the base, while inner HCs survive (arrow heads in a’ and a’). No MyoVIIa+ cells are seen in DTR mice given DT (c’ and d’). Abundant Sox2 staining demonstrates that SCs are well preserved 10 days after DT-induced HC ablation and adenovirus vector inoculation. SCs were also co-labeled with tdTomato (tdTom), demonstrating transfection by the vector (c’’ and d’’). Representative images of 3 biological replicates for each group. Scale bar = 100 μm.

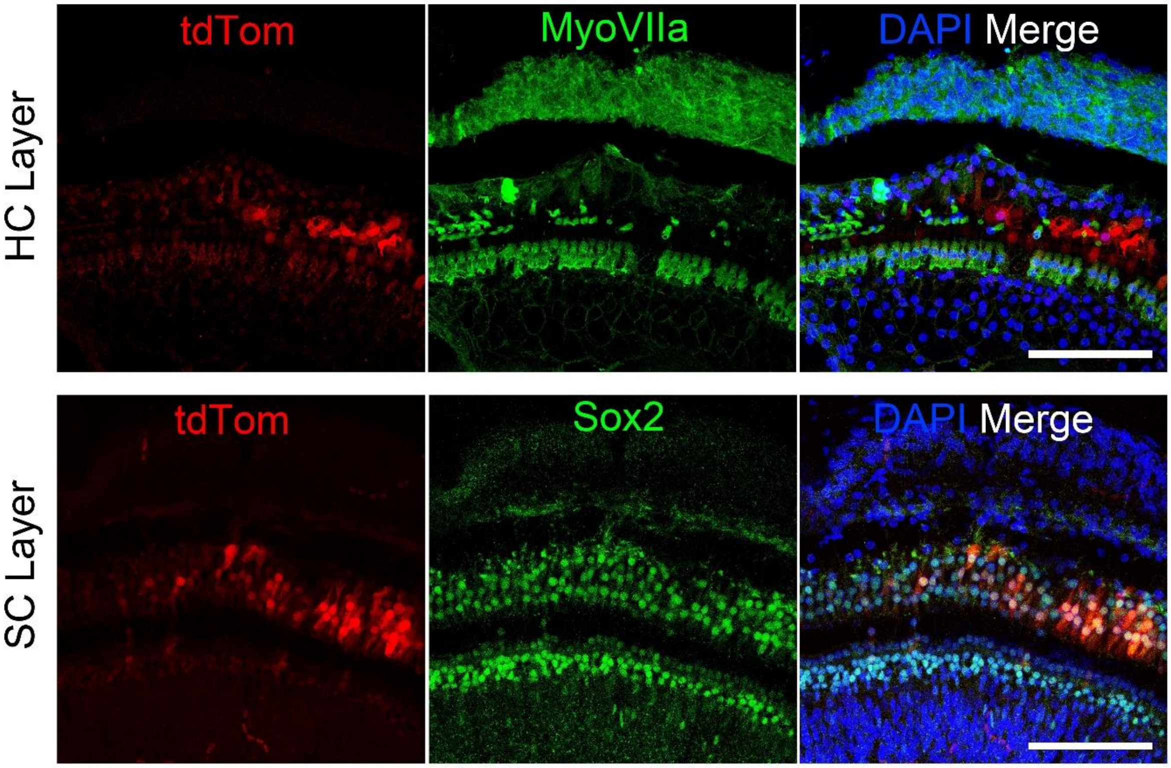

Supplementary Fig. 5. Specific transgene delivery to supporting cells by adenovirus scala media injection

Apical turn of cochleae examined 4 days after injecting adenovirus into non-deafened WT mice show transgene (tdTomato, tdTom) expression co-localized with the SC marker Sox2 and not with the hair cell (HC) marker Myosin VIIa. Representative images of 3 biological replicates. Scale bar = 100 μm.

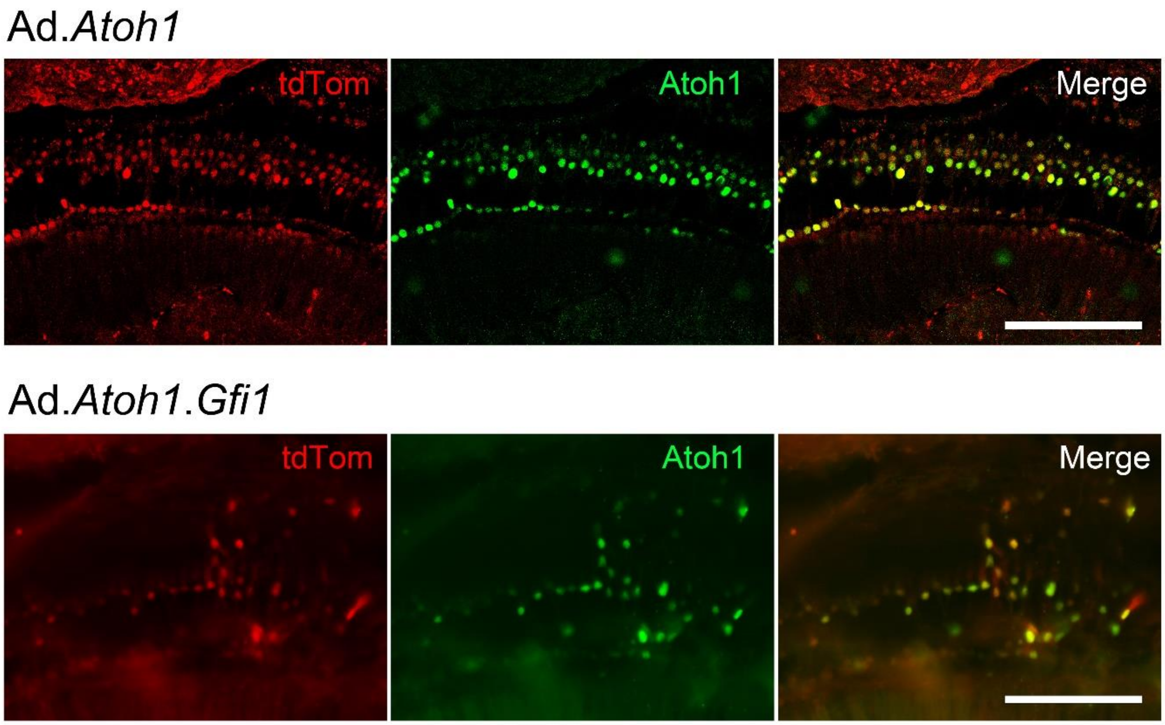

Supplementary Fig. 6. *Atoh1* expression by adenoviral vectors

Cochleae of adult mice four days after injecting adenovirus vectors show robust co-expression of tdTomato with both Ad.*Atoh1* (no *Gfi1*) and Ad.*Gfi1.Atoh1* (with *Gfi1*). tdTom, tdTomato. Scale bar = 100 μm.

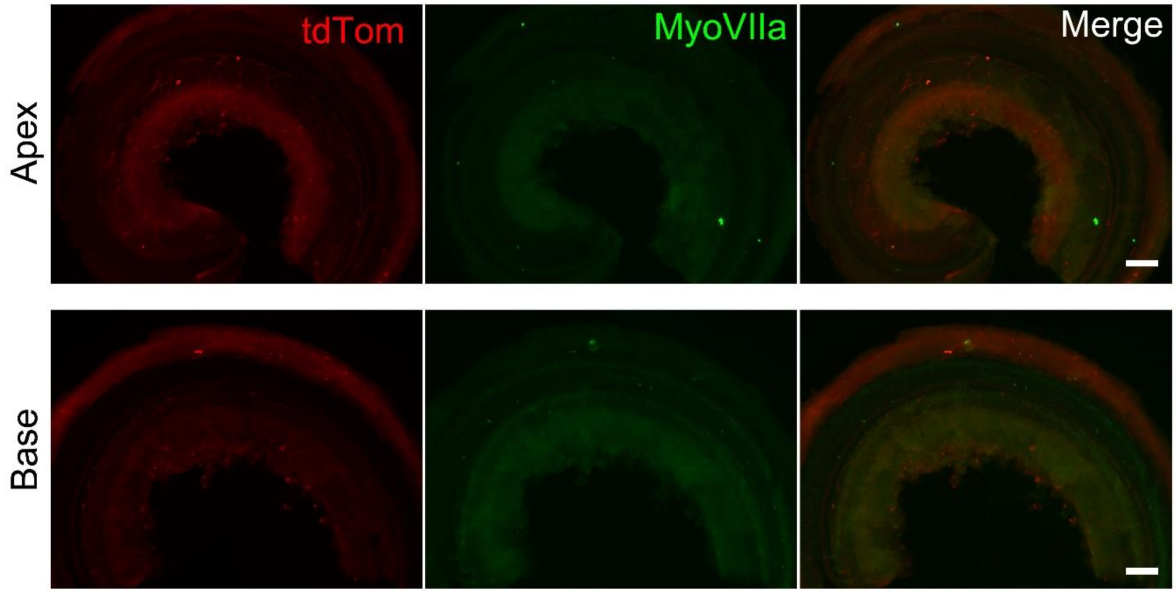

Supplementary Fig. 7. No new hair cells in the contralateral non-injected ear

Contralateral ears of DTR mice at 4 weeks after DT-induced HC ablation and injection of adenovirus into the left ear show no expression of tdTomato or *Atoh1* transgenes in the apex and base, throughout the cochlear duct. Representative images are shown from of 5 biological replicates. tdTom, tdTomato; MyoVIIa, Myosin VIIa. Scale bars = 100 μm.

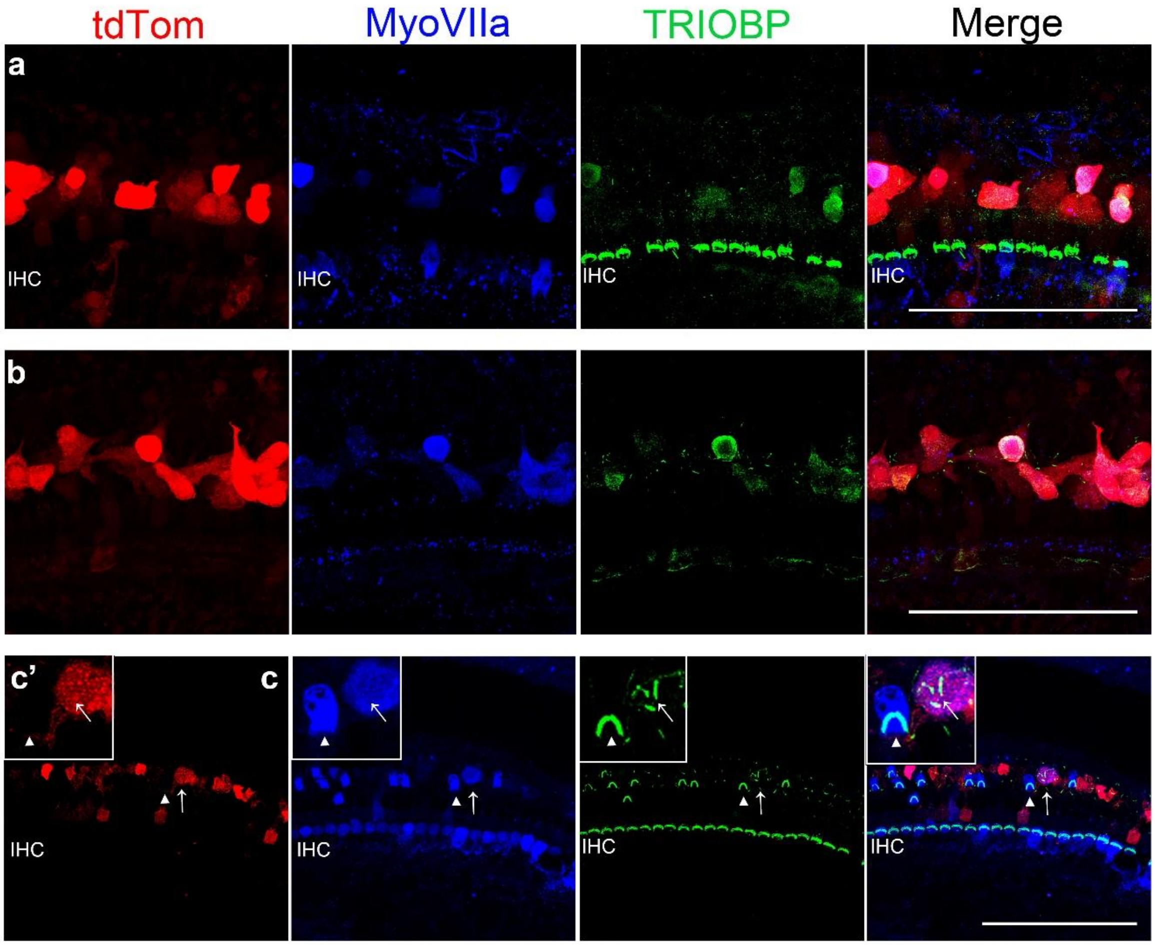

Supplementary Fig. 8. TRIOBP expression in regenerated HCLC.

Eight-weeks after Ad.*Gfi1.Atoh1* injection surgery. In non-deafened mice (a), surviving native cells that were not transfected (tdTomato-negative) show normal arrangement of TRIOBP. In contrast, TRIOBP is widely distributed in the cytoplasm of transfected and converted, similar to tdTomato and Myosin VIIa. The latter pattern is also seen in HCLCs in DT ablated mice (b). Some HCLCs have more concentrated, streak-like distributions of TRIOBP (c and c', arrows). Remaining native OHC shows normal V-shaped arrangement (c and c', arrow heads). Representative images from 3 biological replicates. IHC, native inner hair cell; tdTom, tdTomato; MyoVIIa, Myosin VIIa. Scale bar = 100 μm.
